# Supplementary material for: The Antiaging Effect of Active Fractions and Ent-11α-Hydroxy-15-Oxo-Kaur-16-En-19-Oic Acid Isolated from Adenostemma lavenia (L.) O. Kuntze at the Cellular Level
Source: Antioxidants (Basel). 2020 Aug 8;9(8):719. doi: 10.3390/antiox9080719 (PMC7464069; doi:10.3390/antiox9080719)

Supplementary Data

Table S1. Intracellular metabolites of yeast cells detected on DMSO and 11αOH-KA (150 µM) treatments

Metabolites	Formula	Apex m/z	RT [min]	Group Area: DMSO	Group Area: 11αOH-KA
Biotin	$C_{10}H_{16}N_2O_3S$	243,18221	5,843	8099537,649	15907229,71
Butoctamide semisuccinate	$C_{16}H_{29}NO_5$	316,21073	0,864	5936133,86	13190225,08
Unknown	$C_{40}H_{60}O_8S$	701,40762	3,638	5242823,124	12032933,31
Unknown	$C_{37}H_{72}N_3O_6P$	685,43308	3,621	5120684,444	11662285,07
Unknown	$C_{37}H_{68}N_2O6$	663,45169	3,615	2664894,761	7930026,045
Unknown	$C_8H_{13}N_60_2$	225,06079	0,813	849843,4547	6282982,396
L-Methionine	$C_5H_{11}NO_2S$	150,05807	0,848	207653,9695	218974,4541
Unknown	$C_3H_3F_3O$	112,01799	6,227	5184047,59	3843030,103
Unknown	$C_51H_{56}ClNO_2$	750,40554	22,11	2520741,652	5112323,004
Betaine	$C_4H_6O_4$	118,08617	0,897	1823012,552	16257710,4
Unknown	$C_40H_{73}NO_8P$	726,46032	3,62	2266712,53	4656809,774
Unknown	$C_{33}H_{44}O_{15}$	680,47875	3,616	2118173,933	4582587,922
Unknown	$C_{28}H_{37}NO_9$	531,2865	1,193	2975619,952	3559058,943
Unknown	$C_{18}H_{24}N_2O_2$	300,19808	5,815	1933843,906	3373251,803
Unknown	$C_{27}H_{44}N_7O_3P$	525,25149	1,208	1712909,718	3343581,608
Unknown	$C_{22}H_{19}N$	297,15237	19,659	1997518,151	3049527,529
L-Proline	$C_5H_9NO_2$	115,00251	0,941	2586840,542	109329,407
Unknown	$C_{29}H_{43}O_4P$	487,29579	1,214	1752697,088	2649402,99
Unknown	$C_8H_{16}N_6O_2$	229,14039	0,886	2239259,799	2585457,103
Unknown	$C_{26}H_{48}N_2O_6$	485,35777	5,838	1689317,83	2551336,53
Unknown	$C_{25}H_{39}N_7O_6$	509,27779	1,196	2194307,103	2337190,38
Unknown	$C_{25}H_{34}N_6O_6S$	547,2337	1,185	1830502,46	2077949,303
Unknown	$C_{11}H_8O$	156,04173	0,9	321385,0552	2076863,562
Unknown	$C_{19}H_{26}O_3$	302,1955	0,831	1176559,833	1912419,353
D(+)Glucose	$C_6H_{12}O_6$	179,05508	0,807	163883,5816	1762633,087

Unknown	C17H27NO4	309,17364	19,825	1678361,406	914041,047
Unknown	C22H45ClN8O4	521,33469	5,85	965793,7173	1648714,652
Leucine	C6H13NO2	130,15886	0,872	981081,2137	1631556,71
5,6-Dihydrothymidine	C10H16N2O5	245,11437	0,89	1170214,881	1610035,214
L-Phenylalanine	C9H11NO2	166,08601	0,863	257761,626	1327504,802
Unknown	C26H45NO8S2	563,20764	1,195	1043918,482	1306885,31
1-Methylguanosine	C11H16N5O5	298,09628	0,897	1037668,215	1085104,62
Unknown	C25H49O12P	572,28643	1,17	1048737,194	825035,7202
L-Glutamic acid	C5H9NO4	148,06013	0,907	461958,7692	1039825,735
Unknown	C18H24O4	304,15365	19,443	1012756,485	1034506,928
Unknown	C54H78N8O6	935,61139	3,633	508145,2508	1030757,12
Unknown	C18H32O16	504,32262	1,203	1016036,565	1009452,017
Unknown	C7H6N2O4	182,03278	5,963	953397,8858	894706,5005
Unknown	C19H19BO3	309,17362	19,708	612443,8335	933623,9444
Myristyl sulfate	C14H30O4S	293,04858	0,806	149351,5716	917150,7644
Unknown	C28H39N2O6P	531,25979	1,166	895216,2564	824187,5842
Unknown	C33H40N2O9	608,4193	2,772	353738,8565	854328,9409
L-Serine	C47H74NO5P	764,53602	3,628	316637,5389	832021,3788
Unknown	C4H8O3	104,10714	0,843	432783,6056	807885,0578
Unknown	C10H22O	158,15366	0,865	579744,6764	758864,324
DL-Lactic acid	C9H18O9	269,08754	0,883	416606,2851	230157,4025
Unknown	C29H32O13	588,26035	1,187	671325,7299	686229,6104
Unknown	C17H31N3O5	357,21384	5,811	541131,7737	639921,2577
Unknown	C53H86N2O10P2	971,56916	1,214	354795,6988	635014,5359
Unknown	C29H42O10	550,3045	1,195	585925,1071	629640,2722
L-Tyrosine	C9H11NO3	182,08096	0,826	269012,5724	622924,5089
Unknown	C6H13NO3	242,92502	0,831	590420,1685	292576,6527
Unknown	C34H39N4O6	599,38938	5,815	391234,2206	550372,3618
Unknown	C18H27NO6	353,1996	19,744	367667,6022	500735,1005
(-)Camphanic acid	C21H35NO8	430,24293	0,893	431774,4743	500166,8945
Unknown	C15H22O3	250,19037	5,865	300140,4324	486476,5375

Unknown	C12H19NO2	209,15302	19,379	474239,016	450813,1703
Unknown	C14H23NO	221,1695	5,838	241477,4213	435542,951
N,N-Diisopropylethylamine (DIPEA)	C8H19N	130,15886	1,078	405460,9845	331917,1299
Unknown	C40H75O10P	747,54214	1,187	215103,3254	398179,4079
Unknown	C29H36O15	624,39338	2,772	210003,5607	382949,0324
DL-Arginine	C6H14N4O2	174,93778	0,835	362503,3788	203738,3175
2-Hydroxyfelbamate	C11H14N2O5	255,09711	0,814	236741,1653	362118,9207
Unknown	C20H31NO7	397,22557	19,873	333004,8616	354999,385
Unknown	C31H38O11	586,43754	2,77	96879,21716	352769,52
Nicotinamide	C6H6N2O	123,05521	0,902	342202,5596	288807,8131
Unknown	C35H69NO9P2	710,45066	1,157	270822,5682	336219,5121
Unknown	C33H44O15	680,47928	24,14	222440,991	277611,1025
Unknown	C21H27N7O14P2	663,45275	24,139	226747,8851	266075,507
Unknown	C9H10O4	182,03278	6,153	254150,3313	258241,971
Unknown	C30H23N3O3	473,34392	19,951	257488,3541	211454,171
Unknown	C29H36O9	528,32269	1,208	193895,8807	233174,8671
N-methylethanolamine phosphate	C3H10NO4P	155,8731	0,846	208902,5184	221384,7645
Unknown	C8H19N	128,95842	0,845	217018,0451	213185,0534
Unknown	C16H16O9	352,32014	20,162	173969,7426	183914,4628
3,5-Dinitro-2-hydroxybenzoic acid	C7H4N2O7	228,1951	0,916	129610,8214	179155,9224
Unknown	C19H19N7O6	441,29671	21,305	152052,6715	131385,0745
Unknown	C34H69NO4	556,44089	19,908	162681,6569	135499,6921
Unknown	C18H23NO7	365,13955	19,786	112687,1483	129584,4937
Choline	C5H13NO	102,12785	0,83	109967,0669	350405,6342
Unknown	C10H12O4	196,94616	0,84	124498,6552	95426,33332
Unknown	C25H35NO4	413,28653	20,237	109855,5641	121126,8124
Unknown	C22H30O5	374,30212	20,161	125870,7181	127079,7114
Unknown	C18H32O6	344,31509	19,94	106987,2687	136782,2202
Unknown	C24H37NO2	371,3148	20,977	74068,67179	79919,72493

Figure S1. LC-MS chromatogram of blanko (acetonitrile and LC-MS water; 1:1; v/v) in negative mode

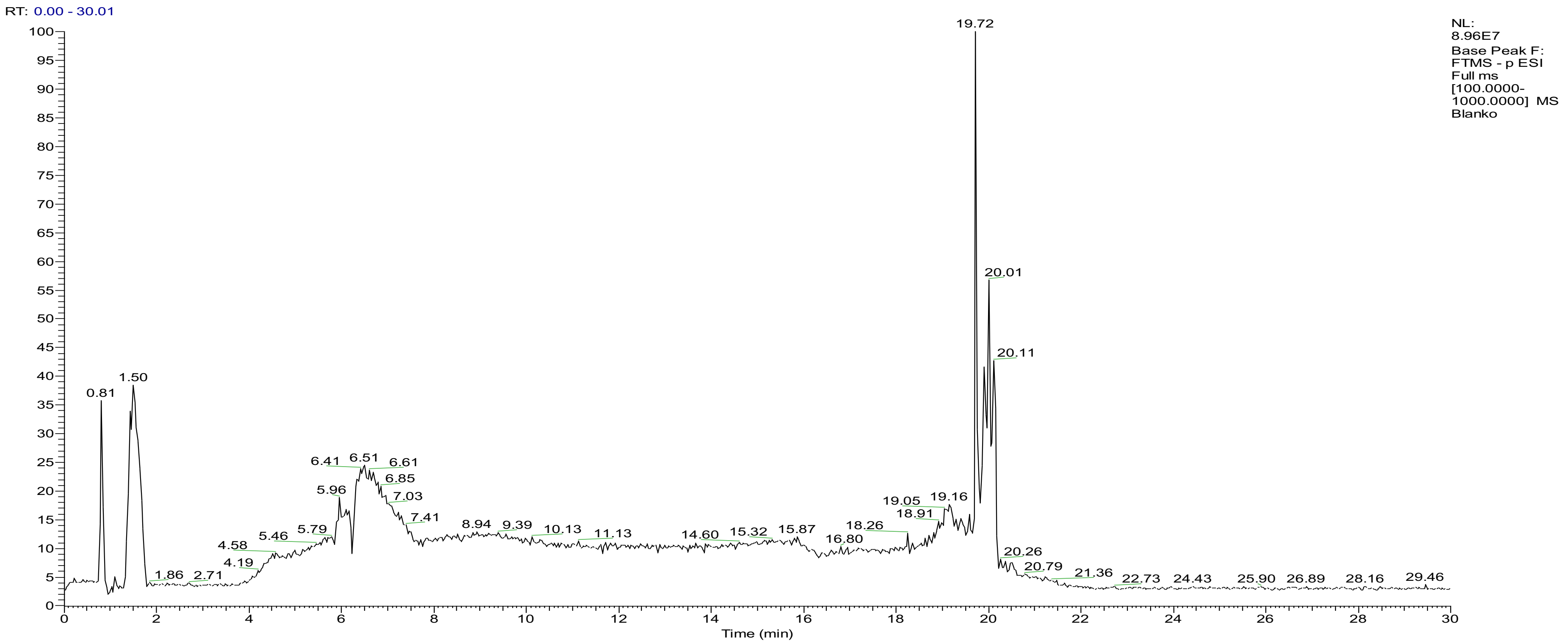

Figure S2. LC-MS chromatogram of DMSO control in negative mode

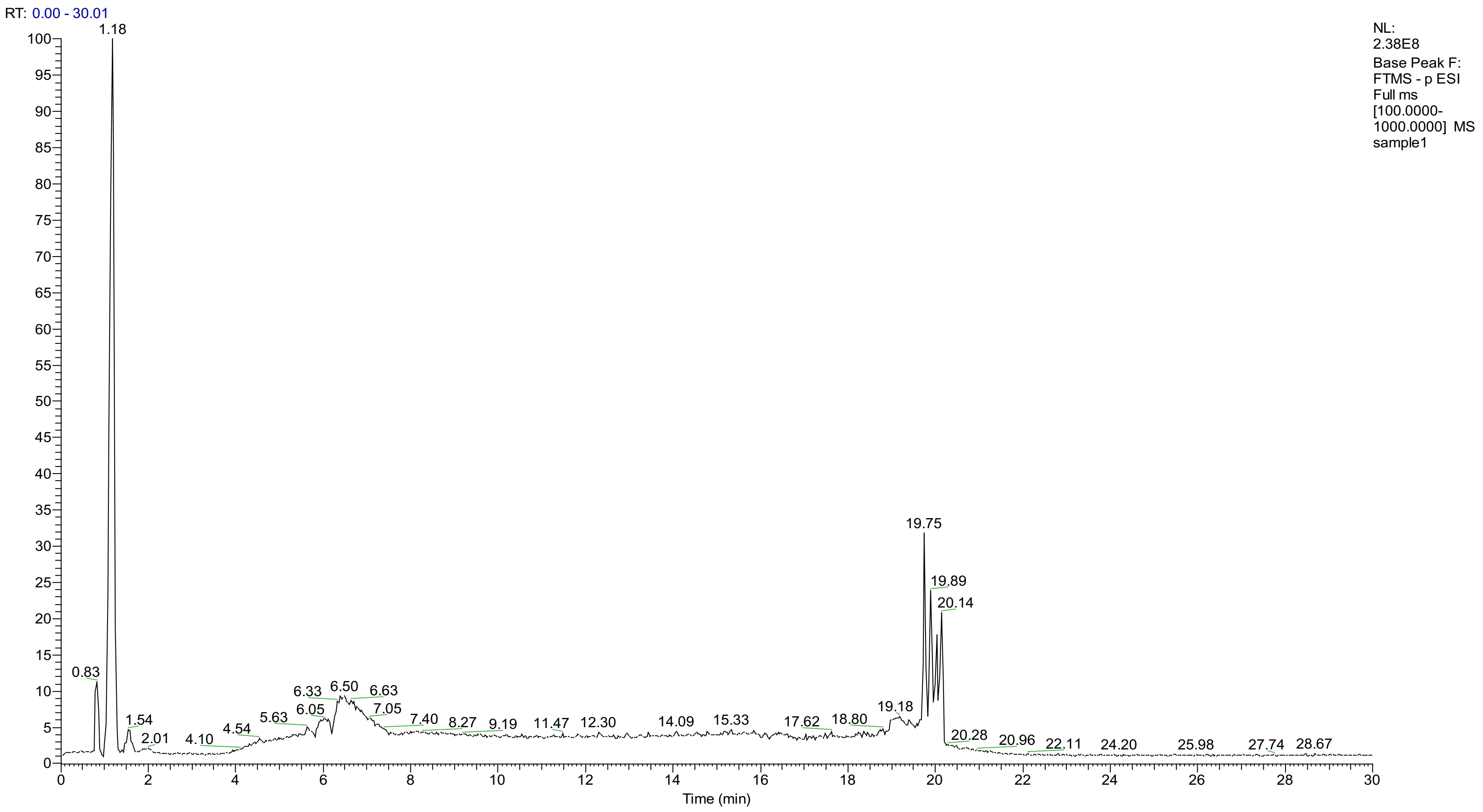
RT: 0.00 - 30.01
NL:
2.38E8
Base Peak F:
FTMS - p ESI
Full ms
[100.0000-
1000.0000] MS
sample1
1.18
0.83
1.54
2.01
4.10
4.54
5.63
6.05
6.33
6.50
6.63
7.05
7.40
8.27
9.19
11.47
12.30
14.09
15.33
17.62
18.80
19.18
19.75
19.89
20.14
20.28
20.96
22.11
24.20
25.98
27.74
28.67
Time (min)

Figure S3. LC-MS chromatogram of 11αOH-KA (150 μM) treatment in negative mode

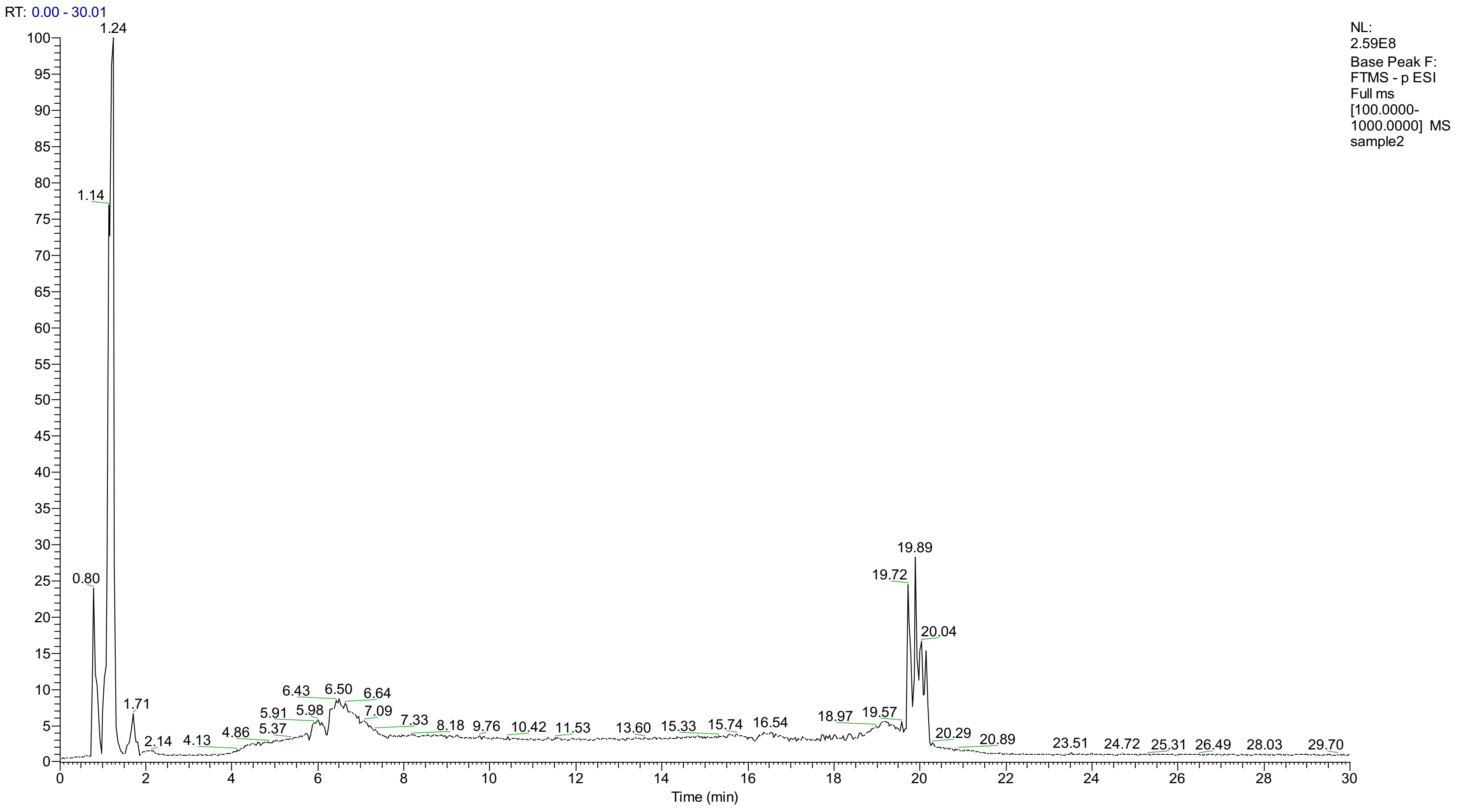

Supplement: Supplementary file 1 [file antioxidants-09-00719-s001.pdf]
